# Supplementary material for: Evaluating Doppel’s impact on Anxiety and Focus amongst adults with ADHD
Source: PLOS Digit Health. 2024 Jul 29;3(7):e0000555. doi: 10.1371/journal.pdig.0000555 (PMC11285955; doi:10.1371/journal.pdig.0000555)
Supplement: Attachment — Submitted filename: Response to reviewers.docx [file pdig.0000555.s001.docx]

The authors claimed to have measured acceptability of the intervention by asking open-ended questions on whether 1) participants had any reasons for not wearing doppel and 2) if they had experienced any problems as a result of wearing the Doppel. I don’t think these accurately measures acceptability of an interventions. I see these questions as more of assessing the participants’ view of the intervention or self-reported barriers in using the intervention and the authors should be clear if that is what they mean.

*Thank you for your comment. Sekhon, Cartwright and Francis (2017) describe acceptability as the extent people delivering or receiving a healthcare intervention consider it to be appropriate based on anticipated or experienced cognitive and emotional responses. It includes components of affective attitude, perceived efficacy and burden. As such, asking about the degree of usage, and reasons for non-adherence provides some of this data. However, we also acknowledge there are additional aspects of acceptability that we have not addressed. This is now reported in the limitations section of the discussion (page 16 line 577).*

Also, the acceptability aspect should be included in the methods section. Only the STAI, ELAS and ASRS scales are included in the self-report survey follow-up and not the interview questions.

*Thank you for this suggestion, this element of the study has now been included in the methods (page 8 line 288).*

Here are some minor comments

1. Spell out the acronym for ADHD

*The acronym has now been spelled out on first use.*

2.	The literature review in the background shows that doppel has been shown to reduce anxiety in previous studies. If the intervention has been shown to reduce anxiety is other studies, then there is high likelihood that the result might be replicated in your study – proving your hypothesis right. To get a compelling argument as to why you need another study on the same intervention, I think the authors should provide a justification as to why this study is needed in the ADHD population.

*Thank you for your feedback. Whilst Doppel has been shown to reduce anxiety in previous studies, these have both been under laboratory conditions at a single time point with a general population sample. The present study is the first to investigate Doppel in a population of young adults with ADHD using Doppel as per it’s user manual. This has now been articulated more explicitly in the study’s rationale (page 6 line 221).*

Reviewer #2: The sample size of 49 participants appears too small for a reliable evaluation of the proposed outcomes. Furthermore, the experiment concluded with a total of 32 participants, without clearly specifying the number of participants in each group (Doppel or comparator). Based on the author's statement that "59.2% of participants reported using medicinal methods, 24.5% reported non-medicinal methods, and 14.3% reported using both," it appears that 73.5% of participants were under some form of medication or intervention. This makes it challenging to accurately assess the effectiveness of the Doppel intervention.

*Thank you for this comment, the number of participants at the 8 week follow up in each condition has now been added to the participants section (page 7 line 229).*

*Moreover, whilst a significant proportion of participants were using some form of treatment for their ADHD during the trial, we feel this is not inconsistent with how Doppel could be used in the real world. Doppel is designed to promote calmness and increase focus rather than as a treatment for ADHD. As such it would likely be used as an adjunct to treatment for additional symptom management. This has now been added to the manuscript (page 16 line 543).*

Reviewer #3: This work presents the results of an 8-week trial of using Doppel, a wearable technology providing vibrations based on the wearer's heart rate.

While the use of digital technology for managing anxiety and increasing focus is motivated well, the work does not adequately review technologies that use bio-feedback such as Doppel does. Doppel has been evaluated previously with a relatively small general population sample in a simulated task with an active and inactive device; this work investigates Doppel with an active device and a comparator device in young adults with ADHD over the course of 8 weeks.

*Thank you for this comment. Additional research focusing on the biofeedback aspect of the technology has now been included in the review (page 6 line 213).*

The protocol was pre-registered with and is decribed relatively well. There are several points to note about the participants: there is a low sample size (not quite meeting the minimum sample size necessary at week 8) and a surprising amount of female participants. Ethical approval was given but is unnecessarily blinded.

*Thank you for this comment. Ethical approval will be unblinded upon publication.*

*The study took place during the COVID 19 pandemic where the UK was in lockdown. We believe this made it more difficult to recruit participants. We also experienced participant drop-out which may have been influenced by the follow ups taking place via survey sent over email. This has now been included in the discussion (page 17 line 571).*

The instruments used to measure anxiety and focus seem appropriate but it is unclear how they were administered and when. The device is decribed well but it was difficult to understand how the active and comparator device differed and why these different setting were implemented in the way they were. At first I thought that the comparator device was providing randomised vibrations but instead it turns out that the vibrations are at static BPM. It seems users could choose which rhythm to use, and I wonder if this had any effect on the measures or how that was accounted for in the analysis. For use, self-reported measures were employed but could this be more objectively measured using app logs?

*Thank you for this comment. The self report measures were administered at baseline, four weeks and eight week time points via a survey link. This detail has now been added to the manuscript (page 7 line 266).*

*The Doppel conditions in the present trial compare the active doppel which has three settings based upon the users resting heart rate and the comparator doppel which has three settings but is not based upon the user’s resting heart rate. This has now been added to the method section (page 6). The reason for this comparator device was because over an 8 week trial duration, given the function of Doppel, it would be very apparent to the user if they were in the control condition if the device gave only intermittent vibrations. This certainly could have impacted the findings and is discussed in the discussion.*

The analysis is fine but I have severe doubts about the robustness of the measures. Similarly, there does not seem to be any difference between active and comparative device in anxienty or focus - wearing either one leads to a decrease in anxiety and an increase in focus over 8 weeks. Hence, I would say that both conditions are a "placebo" in a way whereas this work suggests that there is "some promising initial evidence as to the potential for direct-to-consumer digital health products to be useful in symptom management amongst young adults with ADHD". I strongly suggest to qualify this, especially given the retail price of Doppel.

*Thank you for this comment. The findings suggest participants in both conditions experienced a reduction in anxiety symptoms during the trial. This is discussed in the discussion in relation to research that shows beneficial effects of haptic stimulation amongst those with ADHD. In line with your recommendation we have changed the statement to the following: Whilst the current study cannot determine a specific mechanism of action, the findings provide some promising initial evidence as to the potential for direct-to-consumer digital health products to be useful in symptom management amongst young adults with ADHD.(page 1)*

--------------------
